# Tumor Tissue-Derived Formaldehyde and Acidic Microenvironment Synergistically Induce Bone Cancer Pain

**DOI:** 10.1371/journal.pone.0010234

**Published:** 2010-04-21

**Authors:** Zhiqian Tong, Wenhong Luo, Yanqing Wang, Fei Yang, Ying Han, Hui Li, Hongjun Luo, Bo Duan, Tianle Xu, Qiliang Maoying, Huangying Tan, Jun Wang, Hongmei Zhao, Fengyu Liu, You Wan

**Affiliations:** 1 Neuroscience Research Institute, Peking University, Beijing, China; 2 The Central Laboratory, Shantou University Medical College, Shantou, China; 3 Department of Integrative Medicine and Neurobiology, State Key Laboratory of Medical Neurobiology, Shanghai Medical College, Fudan University, Shanghai, China; 4 Institute of Neuroscience and National Key Laboratory of Neuroscience, Chinese Academy of Sciences, Shanghai, China; 5 Department of TCM Oncology, China-Japan Friendship Hospital, Beijing, China; 6 Department of Thoratic Surgery, Peking University People's Hospital, Beijing, China; 7 Department of General Surgery, Peking University Third Hospital, Beijing, China; 8 Key Laboratory for Neuroscience, Ministry of Education/Ministry of Public Health, Beijing, China; Duke University, United States of America

## Abstract

**Background:**

There is current interest in understanding the molecular mechanisms of tumor-induced bone pain. Accumulated evidence shows that endogenous formaldehyde concentrations are elevated in the blood or urine of patients with breast, prostate or bladder cancer. These cancers are frequently associated with cancer pain especially after bone metastasis. It is well known that transient receptor potential vanilloid receptor 1 (TRPV1) participates in cancer pain. The present study aims to demonstrate that the tumor tissue-derived endogenous formaldehyde induces bone cancer pain via TRPV1 activation under tumor acidic environment.

**Methodology/Principal Findings:**

Endogenous formaldehyde concentration increased significantly in the cultured breast cancer cell lines *in vitro*, in the bone marrow of breast MRMT-1 bone cancer pain model in rats and in tissues from breast cancer and lung cancer patients *in vivo*. Low concentrations (1∼5 mM) of formaldehyde induced pain responses in rat via TRPV1 and this pain response could be significantly enhanced by pH 6.0 (mimicking the acidic tumor microenvironment). Formaldehyde at low concentrations (1 mM to 100 mM) induced a concentration-dependent increase of [Ca^2+^]i in the freshly isolated rat dorsal root ganglion neurons and TRPV1-transfected CHO cells. Furthermore, electrophysiological experiments showed that low concentration formaldehyde-elicited TRPV1 currents could be significantly potentiated by low pH (6.0). TRPV1 antagonists and formaldehyde scavengers attenuated bone cancer pain responses.

**Conclusions/Significance:**

Our data suggest that cancer tissues directly secrete endogenous formaldehyde, and this formaldehyde at low concentration induces metastatic bone cancer pain through TRPV1 activation especially under tumor acidic environment.

## Introduction

Cancer pain is a severe clinical condition, and about 75∼90% of advanced or terminal cancer patients experience chronic pain related to treatment failure and/or tumor progression or metastasis. Malignant bone tumors occur in patients with primary bone cancer, but are far more commonly found to be distant metastases from other primary cancers, notably breast, lung and prostate cancers. As such, bone is the most common site of origin of chronic pain in patients with metastatic lung, breast and prostate cancers and myeloma [1]. In the development of cancer, it is suggested that tumor tissues secrete different kinds of factors including cytokines such as TNF-α and IL-1 [2].

Clinical data have shown that formaldehyde concentration is elevated (2∼8 fold) in the urine of patients with prostate and bladder cancer [3] and in the expired air from tumor-bearing mice and breast cancer patients [4]; and these patients frequently suffer from bone cancer pain [5], [6]. Formaldehyde is considered to be a risk factor of cancer development [7], but for the most part knowledge about formaldehyde secretion by tumor tissue is limited. Whether excessive endogenous formaldehyde induces cancer pain still remains to be determined.

A recent report has indicated that formaldehyde (>0.013 mM) can elicit currents via transient receptor potential vanilloid receptor 1 (TRPV1) and this current could be blocked by the specific TRPV1 antagonist capsazepine in dorsal root ganglion (DRG) neurons [8]. Furthermore, formaldehyde (>0.02 mM) can induce Ca^2+^ influx via TRPV1 and transient receptor potential ankyrin 1 (TRPA1) in transfected-CHO cells. TRPA1 is more sensitive to formaldehyde than TRPV1 [9], [10], however, TRPA1 is sensitive to an intracellular alkalization, not an acidic microenvironment (pH<6.0) [11]. It is well known that tumor tissues typically exist in an acidic microenvironment in the range of pH 4∼5 [12]. TRPV1 is a cation channel activated by capsaicin, noxious heat, low pH (pH<5.5) and endogenous vanilloids [13], [14]. More importantly, TRPV1-mediated currents induced by capsaicin, endogenous vanilloids and ethanol can be enhanced by low pH [15]. TRPV1 participates in nociception especially under acidic conditions [16] and is considered to play an important role in cancer pain [17]. Clinical investigation found TRPV1 over-expression in patients with pancreatic carcinoma [18], bladder cancer [19] and breast cancer [20], and such an over-expression is positively correlated with the intensity of pain [18]. Therefore, in the present study, we postulated that excessive cancer tissue-derived endogenous formaldehyde induces bone cancer pain via TRPV1 especially under an acidic tumor microenvironment.

## Results

### Formaldehyde concentration increased in cultured cancer cell lines and tumor tissues from cancer patients

**Figure 1:**
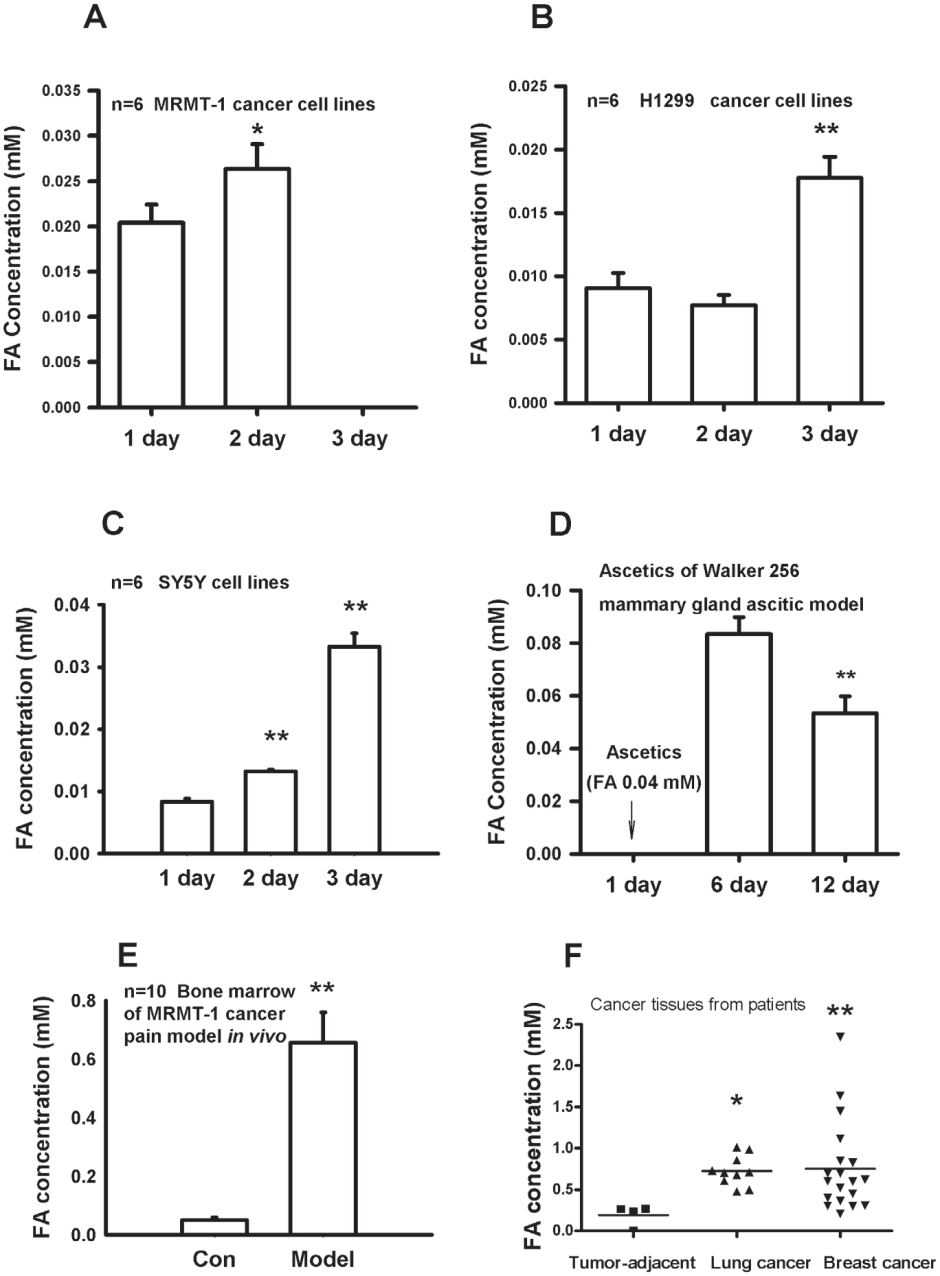
Formaldehyde concentration in the cultured cancer cell lines *in vitro* and *in vivo*. (A) MRMT-1 cancer cells. (B) Human H1299 lung cancer cells. (C) Human SY5Y cancer cells. (D) Ascites from peritoneal inoculation of Walker 256 mammary gland carcinoma cells. (E) Bone morrow of MRMT-1 breast cancer pain model rat *in vivo*. (F) Tumor tissues from lung and beast cancer patients. * *p<*0.05, ^**^ *p<*0.01, compared with that of the first day.

We first investigated whether formaldehyde concentration was elevated in cultured tumor cell lines *in vitro*. Formaldehyde concentrations in rat breast cancer cell line MRMT-1 cells were significantly higher on day 2 than those of controls on the first day after cell density reached 10^5^ cells/ml and 8×10^5^ cells/ml respectively, but decreased on day 3 when the cell density decreased (n = 6) (Fig. 1A). Formaldehyde concentration was also significantly increased in human lung cancer cell line H1299 cells and SY5Y cells (Fig. 1, B and C). Syngeneic Walker 256 mammary gland carcinoma cells were cultured by seeding into the abdominal cavity. A significant increase in the formaldehyde concentration was found in 0.5 ml ascitic fluid (2×10^7^ cells/ml) 6 d and 12 d after inoculation. Formaldehyde concentration was elevated two fold on day 6 after inoculation (from 0.04 mM to 0.08 mM) and decreased on day 12 when tumor cells grew into terminal phase. The highest concentration was 0.10 mM (Fig. 1D). Formaldehyde concentration was significantly elevated in bone marrows of MRMT-1 cancer pain model as compared with that in normal bone marrow (Fig. 1E). These results indicate that the formaldehyde concentration was elevated in all tested tumor cell lines.

Pain visual analogue scores (VAS) were used in groups of patients with (n = 19) and without (n = 6) breast pain. Nineteen patients had significantly suffered from cancer pain. Only 1 patient reported thermal pain descriptors (burning, hot), while most reported ache and tenderness. VAS scores in these patients and controls were 3.8±0.3 and 0.3±0.2 respectively. Formaldehyde concentration was examined in tumor tissues from patients (Fig. 1F). In preparations from lung cancer patients, the average formaldehyde concentration was 0.72±0.06 mM (n = 10) with the highest concentration 1.01 mM. This was significantly higher than that in the normal tissues adjacent to the cancer (0.19±0.06 mM). In breast cancer tissues from patients, the formaldehyde concentration was 0.75±0.12 mM with the highest concentration 2.35 mM. Although the breast tumor adjacent tissues (as controls) were not gained, levels of formaldehyde in human tissues were approximately 0.1∼0.2 mM as previously reported [21]. These levels are similar to the average level (0.19±0.06 mM) found in human lung cancer adjacent tissues in the present experiment. Taken together, these data show that the tumor-derived formaldehyde concentration is elevated in cancer tissues, strongly suggesting that tumor tissues secrete formaldehyde.

### Formaldehyde scavengers and TRPV1 antagonists attenuated formalin-induced pain behaviors

**Figure 2:**
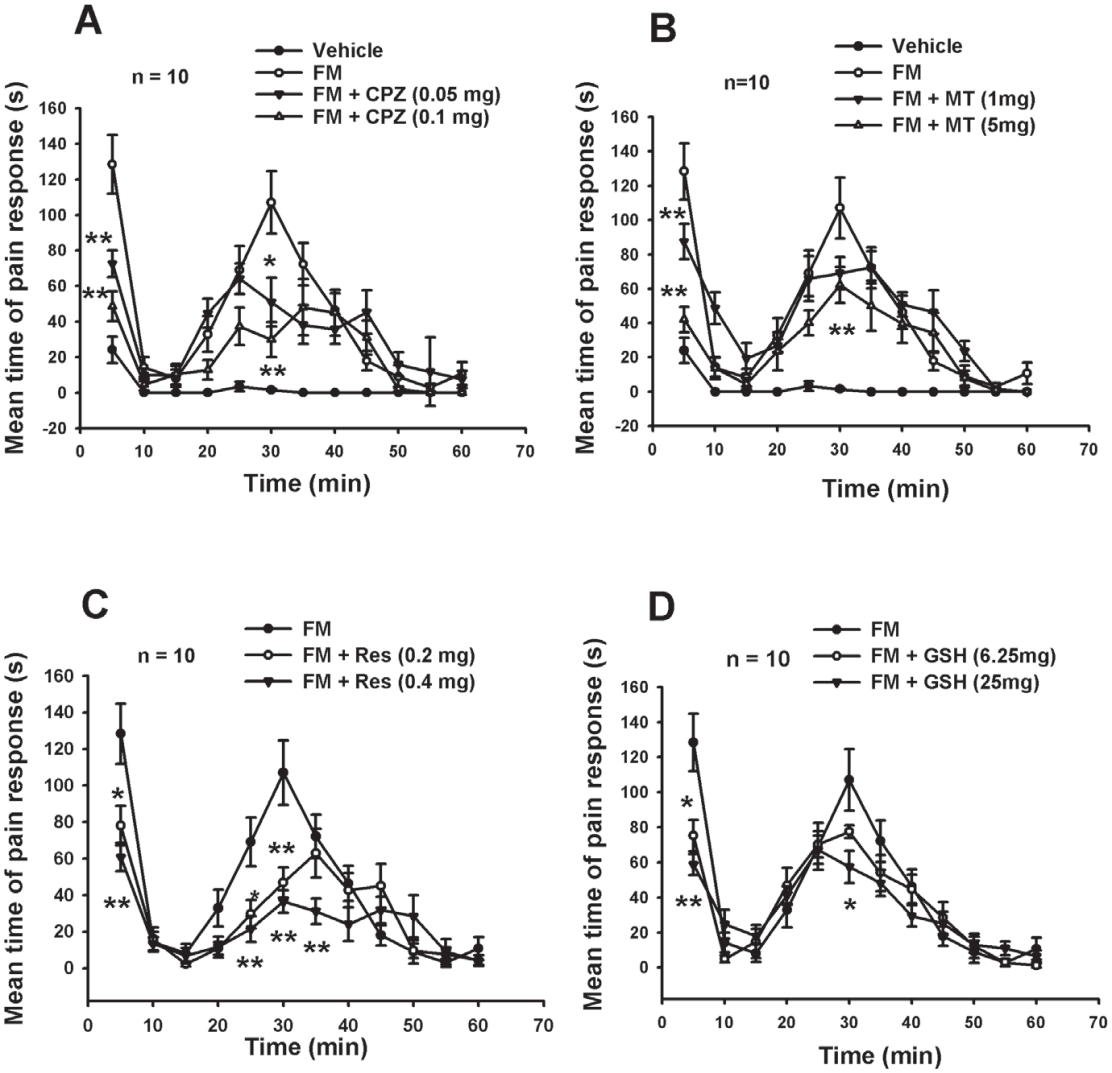
TRPV1 antagonists and formaldehyde scavengers inhibited formalin-induced pain response in rats. (A) Capsazepine (CPZ, a TRPV1 antagonist); (B) Melatonin (MT); (C and D) Formaldehyde scavengers: Resveratrol (Res) and Glutathione (GSH). mg/paw. * *p<*0.05, ^**^ *p<*0.01, compared with the formaldehyde injection groups. n = 10.

The formalin test (5% formalin, i.e. 1662 mM formaldehyde) is a classic pain model commonly used to evaluate analgesic medicines. We found that formaldehyde scavengers glutathione (GSH) and resveratrol (Res), and TRPV1 antagonists capsazepine (CPZ) and melatonin (MT) significantly decreased the number of flinchings in a dose-dependent manner in both acute and tonic phases (Fig. 2, A–D), similar to that in previous reports [1], [22]. The solvent used for these regents, DMSO (final concentration <10%) by itself did not show significant effect (Fig. 2A).

### Low concentration formaldehyde-induced pain behaviors via TRPV1 was enhanced by low pH

**Figure 3:**
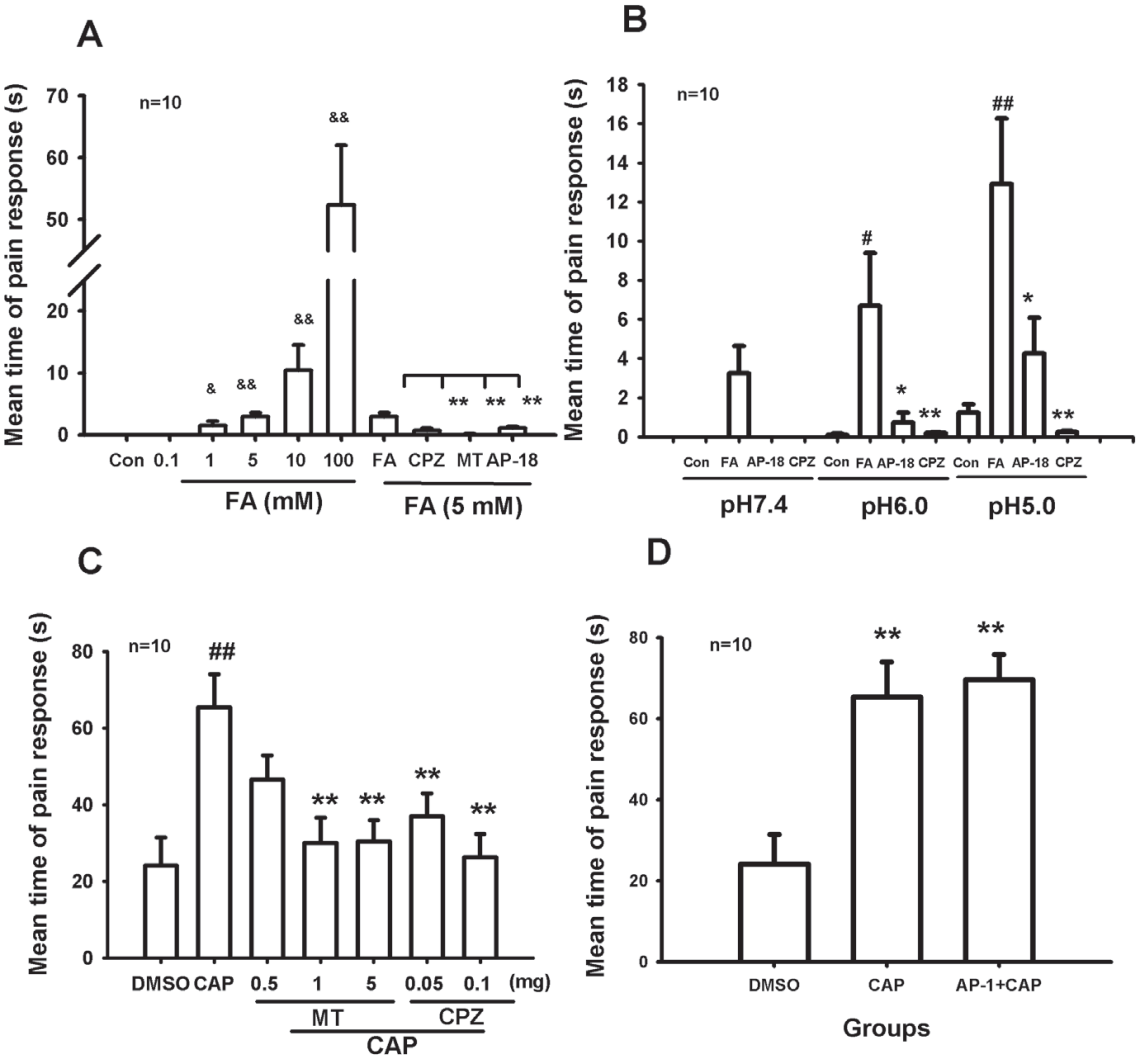
Capsaicin- or formaldehyde (100 µl/paw)-induced acute pain responses. (A) Melatonin, capsazepine and AP-18 attenuated formaldehyde-induced pain responses. (B) Low pH enhanced formaldehyde-induced pain responses. (C) Melatonin (MT) and capsazepine (CPZ) blocked capsaicin (0.5 mM)-induced pain responses. (D) AP-18 did not block capsaicin-induced pain responses. Con: control; DMSO: vehicle; CAP: capsaicine. * *p<*0.05, ** *p<*0.01, ^##^ *p<*0.01, ^&^ *p*<0.05, ^&&^ *p*<0.01, all compared with respective controls. n = 10.

Formalin 5% functioning as a chemical irritant can induce nociceptive behavioral responses (pain). This in turn raises an interesting question of whether formaldehyde at pathologically low concentrations (1∼3 mM, based on the concentrations of formaldehyde detected in human tumor tissues) can induce pain responses, and whether TRPV1 or TRPA1 is involved in the pain responses. Intraplantar injection of formaldehyde (0.1 mM to 100 mM) to the right hind paw evoked dose-dependent, short-lasting (5 min) pain responses of the injected paw in normal rats. Capsazepine, melatonin and AP-18 (a TRPA1 antagonist) all attenuated the low concentration formaldehyde (5 mM)-induced pain responses (Fig. 3A). These results indicate that formaldehyde at low pathological concentration can induce pain behavioral responses, possibly through activation of TRPV1 and TRPA1.

In addition, formaldehyde (3 mM) with a low pH of 5.0 or 6.0 (mimicking the acidic cancer microenvironment) induced more severe pain responses than formaldehyde in a neutral environment (pH 7.4). These responses were partially inhibited by AP-18, but almost completely inhibited by capsazepine (a TRPV1 antagonist) (Fig. 3B). Moreover, capsazepine and melatonin attenuated capsaicin-induced pain responses (Fig. 3C), but AP-18 did not (Fig. 3D). This result is similar to a previous report [23]. These data suggest that TRPV1, but not TRPA1, plays a key role in low concentration formaldehyde-induced pain behaviors under acidic environment.

### Formaldehyde induced Ca^2+^ influx in dorsal root ganglion (DRG) neurons and TRPV1-CHO cells *in vitro*

**Figure 4:**
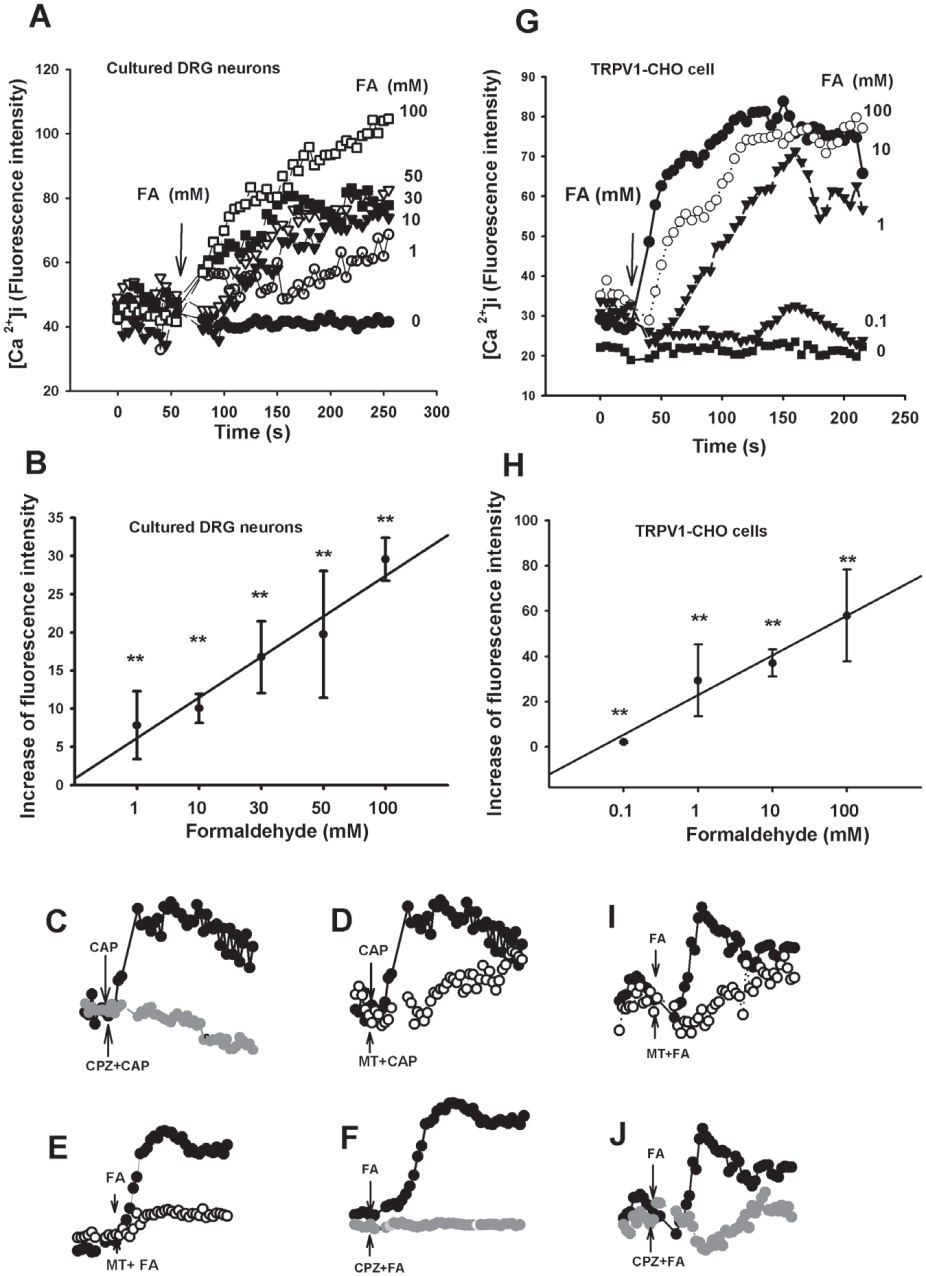
Formaldehyde-induced increase in cytosolic [Ca^2+^]i in cultured DRG neurons and in TRPV1-CHO cells. (A) Formaldehyde-induced dose-dependent increase of cytosolic [Ca^2+^]i in DRG neurons. (B) Statistical analysis of formaldehyde-induced [Ca^2+^]i influx in DRG neurons. (C and D) Inhibition of capsazepin (CPZ, 100 µM) and melatonin (MT, 200 µM) on formaldehyde-induced [Ca^2+^]i influx in DRG neurons. (E and F) Inhibition of MT and CPZ on capsaicin (CAP, 10 µM)-induced Ca^2+^ influx in DRG neurons. (G) Formaldehyde-induced dose-dependent increase of cytosolic [Ca^2+^]i in TRPV1-CHO cells. (H) Statistical analysis of formaldehyde-induced Ca^2+^ influx in TRPV1-CHO cells. (I and J) Inhibition of MT and CPZ on formaldehyde-induced Ca^2+^ influx in TRPV1-CHO cells. ^**^ *p<*0.01, compared with controls. n = 5∼10.

We further used calcium imaging to test whether low concentration formaldehyde can directly excite DRG neurons via TRPV1. As expected, formaldehyde at concentrations of 1 mM to 100 mM induced a concentration-dependent increase of [Ca^2+^]i in freshly isolated rat DRG neurons (Fig. 4, A and B). TRPV1 antagonists capsazepine and melatonin inhibited the [Ca^2+^]i increase evoked by formaldehyde (Fig. 4, C and D) or by capsaicin (Fig. 4, E and F).

To verify whether formaldehyde directly activates TRPV1, we next examined the effect of formaldehyde on TRPV1-transfected CHO (TRPV1-CHO) cells. As expected, formaldehyde (>0.1 mM) induced an increase of cytosolic [Ca^2+^]i in a concentration-dependent manner (Fig. 4, G and H). As a control, in the untransfected CHO cells, formaldehyde at 100 mM elicited only slight Ca^2+^ influx (data not shown). Formaldehyde induced Ca^2+^ influx in the TRPV1-CHO cells was significantly inhibited by the TRPV1 antagonist capsazepine and melatonin (Fig. 4, I and J).

### Formaldehyde and pH 6.0 synergistically elicited currents in TRPV1-CHO cells *in vitro*

**Figure 5:**
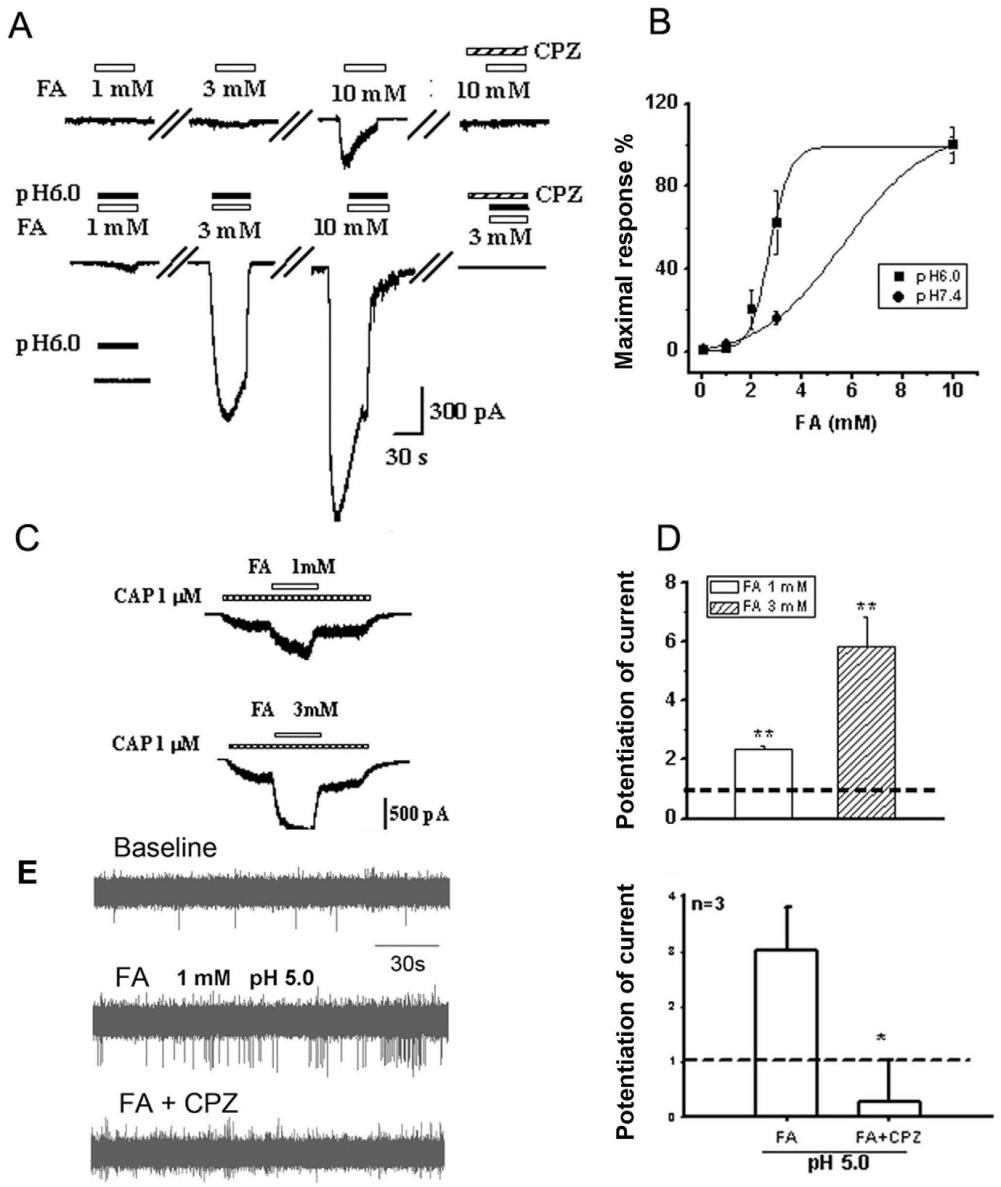
Enhancement of low pH on formaldehyde- or capsaicin-induced TRPV1 currents in TRPV1-CHO cells *in vitro* and formaldehyde-induced C-fiber discharges *in vivo*. (A) Formaldehyde (FA)-induced currents and pH 6.0 enhancement on the currents with patch clamp recording. TRPV1 antagonist capsazapine (CPZ) inhibited both the formaldehyde-induced currents and the pH 6.0 enhancement. (B) Statistical results of low pH enhancement of formaldehyde-induced currents. (C) Formaldehyde enhancement on capsaicin (CAP)-induced currents. (D) Statistical results of formaldehyde enhancement on capsaicin-induced currents. n = 6∼10. (E) Formaldehyde-induced C-fiber discharges under an acidic environment (pH 5.0) with extracellular recording in normal rats. The discharge was inhibited by CPZ. (F) Statistical results of CPZ inhibition on the formaldehyde-induced C-fiber discharges. * *p*<0.05, ** *p*<0.01. n = 3.

Since nociceptive behaviors (pain) induced by formaldehyde at pH 6.0 were sensitive to the TRPV1 antagonist, formaldehyde at pH 6.0 may act directly on TRPV1 (Fig. 3B). We recorded the TRPV1 current induced by capsaicin and formaldehyde (with or without pH 6.0) using patch clamp recording in TRPV1-CHO cells. Capsaicin at 10 µM induced an inward current with voltage clamped at −60 mV. Capsazepine, a TRPV1 antagonist, strongly suppressed the capsaicin-induced current. Similarly, formaldehyde at 3 mM (concentration detected in human tumor tissues) induced an inward current in TRPV1-CHO cells in a concentration-dependent manner and 10 µM capsazepine blocked the formaldehyde-induced current (Fig. 5A). As controls, neither 3 mM formaldehyde, nor 10 µM capsaicin, nor formaldehyde plus capsaicin induced any current in the untransfected CHO cells (data not shown).

Although low pH of 6.0 in extracellular solution had little effect on TRPV1-CHO cells, currents induced by formaldehyde at 1∼10 mM were significantly potentiated by pH 6.0 (Fig. 5, A and B). This result indicates that there is a synergistic effect between formaldehyde and an acidic environment. As a positive control, formaldehyde at 1 and 3 mM also markedly potentiated capsaicin (1 µM)-induced current in the TRPV1-CHO cells (Fig. 5, C and D). These data suggest that formaldehyde directly activates TRPV1 with more efficiency at low pH.

### Formaldehyde and pH 5.0 synergistically elicited C-fiber discharges via TRPV1 *in vivo*

Of greater importance, we tried to determine whether formaldehyde within the concentration range detected in the cancer tissues from patients is functional in exciting peripheral nociceptive nerve fibers. Peripheral C-fibers transduce nociceptive information to conduct nociceptive information. An increase in nociceptive C-fiber firings is a common characteristic of pain. Since formaldehyde at pH 5.0 (mimicking an extremely acidic tumor microenvironment) induced more severe pain behaviors than pH 5.0 alone (Fig. 3B), we tested whether formaldehyde can excite C-fibers. It was found that formaldehyde (1 mM, similar to the levels detected in human lung cancer tissues) at pH 5.0 can directly excite C-fibers. The number of action potentials increased significantly after formaldehyde (under pH 5.0) injection into the receptive field of the C-fibers. This C-fiber excitation could be blocked by the TRPV1 antagonist capsazepine (CPZ) (Fig. 5, E and F).

### Formaldehyde scavengers inhibited formaldehyde-induced neurotoxicity in cultured DRG neurons

**Figure 6:**
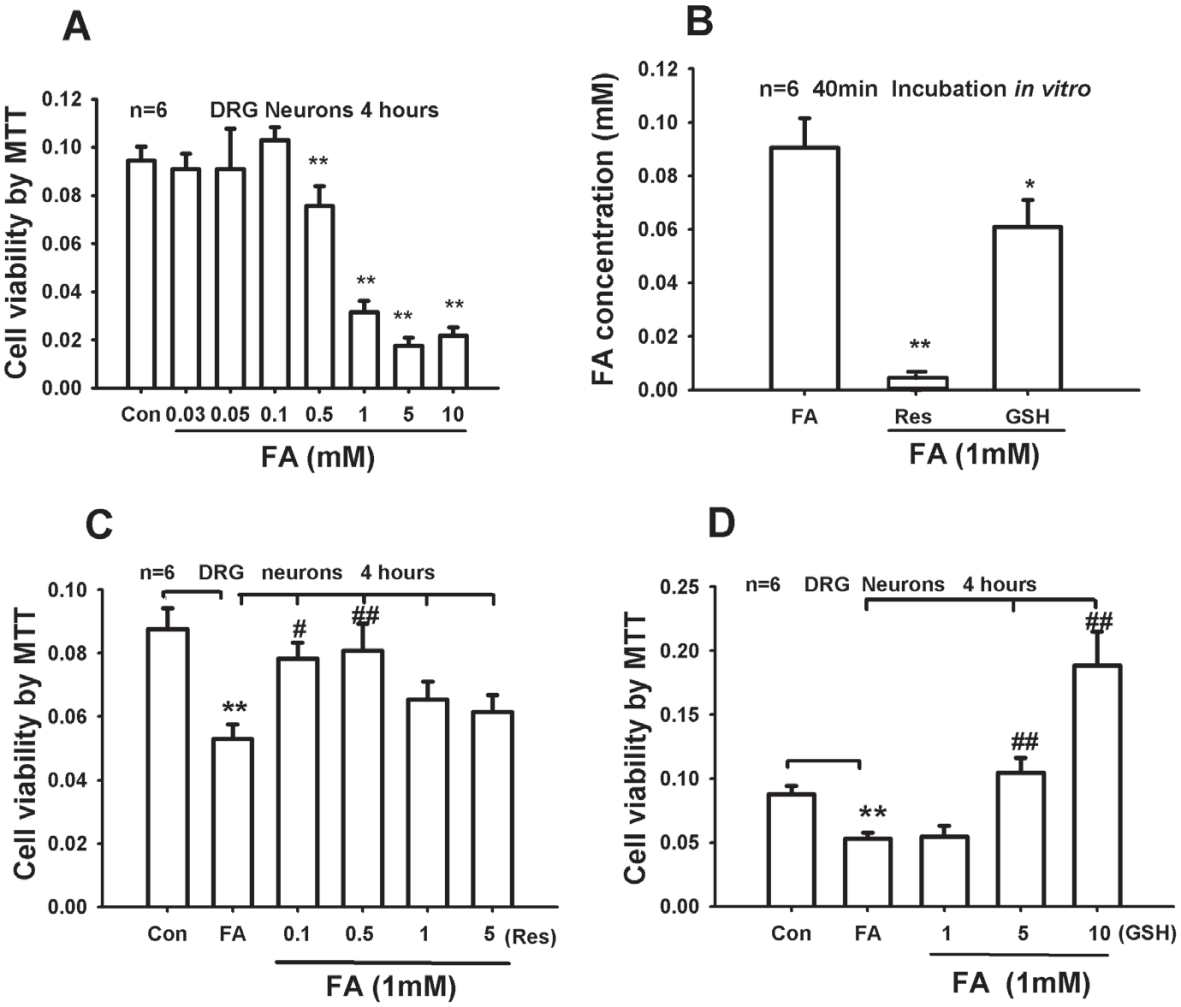
Inhibition of formaldehyde scavengers on formaldehyde-induced neurotoxicity. (A) Formaldehyde (FA) decreased cell viability of the cultured DRG neurons with a dose-dependent manner. (B) Chemical reaction of formaldehyde with resveratrol and glutathione. (C) Resveratrol (Res) and (D) glutathione (GSH) inhibition on the formaldehyde-induced cell viability decrease. * *p<*0.05, ** *p<*0.01, ^#^ *p<*0.05, ^##^ *p<*0.01. n = 6.

We further tested the neurotoxicity of formaldehyde within the above-measured concentration range (>0.1 mM) in the cultured DRG neurons. It was shown that formaldehyde was neurotoxic to DRG neurons in a dose-dependent manner (Fig. 6A). Resveratrol, an exogenous formaldehyde scavenger, and glutathione, an endogenous formaldehyde scavenger, showed spontaneous chemical interaction with formaldehyde in PBS solution within 40 minutes (Fig. 6B). These formaldehyde scavengers decreased formaldehyde-induced neurotoxicity with a concentration-dependent manner (Fig. 6, C and D).

### Formaldehyde scavengers and TRPV1 antagonists attenuated bone cancer pain behaviors in rats

**Figure 7:**
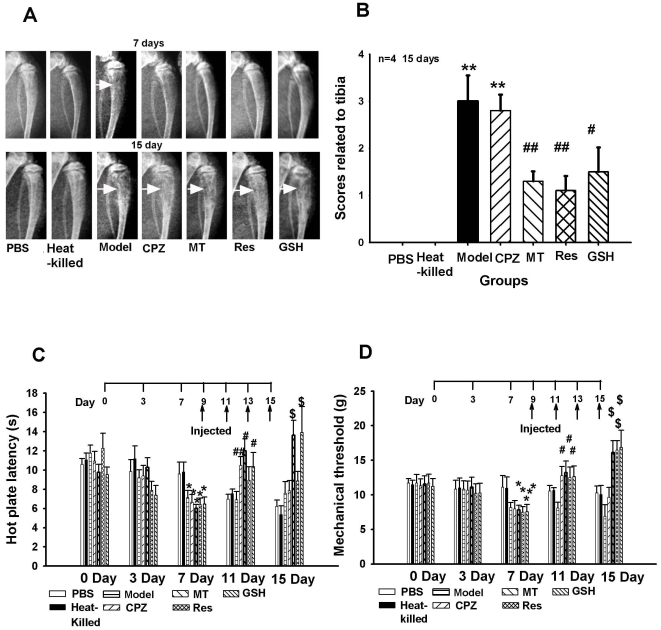
Inhibition of TRPV1 antagonists or formaldehyde scavengers on rat MRMT-1 bone cancer pain behaviors. (A) Radiological confirmation of tumor development in the tibia of MRMT-1 pain model rats. (B) Scores related to the tibia (bone) in different treatment groups. n = 4. (C) Thermal hyperalgesia. Formaldehyde scavengers resveratrol (Res, 0.4 mg/ml) and glutathione (GSH, 25 mg/ml), TRPV1 antagonists capsazepine (CPZ, 0.1 mg/ml) and melatonin (MT, 5 mg/ml) increased hot plate latency. (D) Mechanical allodynia. Res, GSH, CPZ and MT increased mechanical threshold. * *p<*0.05, ** *p<*0.01; ^#^ *p<*0.05, ^##^ *p<*0.01, ^$^ *p*<0.05, compared with respective PBS groups. n = 10. Killed: heat-killed group.

As shown in Fig. 7A, X-ray revealed that no radiological change (score  = 0) was found in animals treated with heat-killed tumor cells or with PBS solution. However, 7 days after injection with MRMT-1 cells, the bone showed some loss of medullary bone and apparent erosion of the cortical bone. Further deterioration was detected on day 15 post-injection with additional full thickness unicortical bone loss. Formaldehyde scavengers (glutathione and resveratrol) and melatonin all significantly decreased bone destruction; however, capsazepine, a TRPV1 antagonist, did not protect bone structure from erosion on day 15 (Fig. 7, A and B). Pain behaviors including thermal hyperalgesia and mechanical allodynia were observed from 7 to 15 days after injection of MRMT-1 cancer cells. It was also found that the pain behaviors were attenuated by capsazepine, melatonin and formaldehyde scavengers (glutathione and resveratrol) on days 11 and 15 after MRMT-1 inoculation (Fig. 7, C and D).

It was of interest to note that even though capsazepine and melatonin both attenuated bone cancer pain behaviors, the formaldehyde concentration in the spinal cord and blood of the cancer pain model rats on days 15 was still kept higher than that of the control rats. More importantly, resveratrol and glutathione (formaldehyde scavengers) inhibited bone cancer pain behaviors by decreasing excessive formaldehyde in the spinal cord (Fig. S1, A and B).

## Discussion

### Tumor tissues directly secrete endogenous formaldehyde

The physiological formaldehyde level was reported to be approximately 0.1 mM in the blood or brain of human and non-human animal [21]. Surprisingly, clinical data showed that formaldehyde concentrations were significantly elevated (2∼8 fold) in urine from patients with bladder cancer and prostate cancer [3], in the expiration of some patients suffering from breast cancer [4] and especially high in blood samples (8∼10 folds) from certain patients with tumor [24]. Formaldehyde was also elevated in lymphocytes in chronic lymphocytic leukemia [7]. The expression and activity of formaldehyde generating enzymes, such as lysine-specific demethylase 1 (LSD1) [25], [26], semicarbazide-sensitive amine oxidase (SSAO) [27], [28] and cytochrome P-450 [29], [30], formaldehyde degrading enzymes, such as aldehyde dehydrogenase 2 (ALDH2) and class III alcohol dehydrogenase (ADH3) [31], [32], are considered to have critical roles in the pathogenesis of breast cancer. Over-expression of ADH3 has been found in cancer tissues, which defenses formaldehyde [33]. This implies that tumor tissues can tolerate formaldehyde at abnormal levels. The present study gives direct evidence that formaldehyde can be secreted from the cultured cancer cell lines in *vitro* and tumor tissues from certain cancer pain patients *in vivo*, and its concentration may reach abnormally high levels (Fig. 1). Because the bone cavity volume of rats is small and the amount of bone marrow tissue is little, four bone marrows from MRMT-1 breast cancer pain models were combined to one tube for HPLC measurement in the present study. A marked elevation of formaldehyde level was found in bone marrow of cancer pain model rats (Fig. 1E). This agrees with a previous report that formaldehyde could be accumulated in bone marrow [34]. Interestingly, formaldehyde level in blood was also obviously elevated in MRMT cancer pain model in rats (Fig. S1, B) and formaldehyde is considered as a cause of cancer [35]. These reports suggest that excessive formaldehyde production by tumor tissues is possibly a critical factor in tissue cancerization or osseous metastasis.

### Cancer tissue-derived excessive formaldehyde induces bone destruction

Cancer cell metastasis to bone marrow increases osteolysis, osteoclastic activity and induces an acidic microenvironment [36]. This is related to the fact that osteoclasts resorb bone by maintaining an extracellular microenvironment of pH 4∼5 [12]. Acidification is a cause of pain in cancer and inflammation [36]. The activated osteoclasts increase proton-induced stimulation of TRPV1 or acid-sensitive ion channels (ASICs) on sensory nerve fibers that innervate bone [1]. Another source of protons is lysis of tumor cells themselves. Cancer cells have a lower intracellular pH than normal cells [37], as solid tumors outgrow their vascular supply, then cancer tissue becomes necrotic, which contributes to the acidic environment [38]. A recent research report also demonstrated that formaldehyde, gradually released by root canal sealers, elicited bone necrosis [39]. Elevated formaldehyde was also observed in patients with dental caries [40]. Cytotoxicity resulting from excessive formaldehyde on human osteoblastic cells has been considered to be an important factor in bone destruction [41], [42]. Formaldehyde can accumulate in bone marrow [34]. In our present study, formaldehyde concentration was elevated to about 0.6 mM in bone marrow of MRMT-1 bone cancer pain model in rats (Fig. 1E). This level is high enough to be toxic to osteoblastic cells. Bone destruction was found in the MRMT-1 bone cancer pain model in rats. Formaldehyde scavengers, resveratrol and glutathione obviously decreased bone destruction in the present study (Figs. 6B, 7A and 7B). Therefore, excessive formaldehyde secreted by cancer tissues may play a role in bone destruction. This bone destruction then contributes to cancer pain, because nerve fiber endings innervating bone is more easily exposed to tumor tissue-derived factors.

### Formaldehyde induces pain responses via TRPV1 and/or TRPA1

Breast, lung and bladder cancer patients frequently suffer from bone cancer pain [5], [6]. In the present study, mechanical allodynia was found in breast cancer pain patients and in the affected hind paw in the MRMT-1 breast cancer pain model rats (Fig. 7, B and C); this result is similar to that observed in a previous report [43]. The MRMT-1 bone cancer pain model is widely used in breast cancer bone pain research [44]. TRPV1 antagonists attenuated endogenous formaldehyde-induced bone cancer pain behaviors (Fig. 7, B and C). The selective TRPV1 antagonists, such as iodo-resiniferatoxin [45] and capsazepine, and the non-selective antagonist ruthenium red [46], inhibited formalin-induced pain behaviors. These findings suggest that TRPV1 may participate in formaldehyde-evoked pain. In our behavior tests, we found that formaldehyde at low pathological (3 mM, based on concentration detected in human cancer tissues) in an acidic environment induced rat pain responses via TRPV1 *in vivo* (Fig. 3B). Capsazepine (a TRPV1 antagonist) attenuated capsaicin- or formaldehyde- (pH 6.0) induced pain responses in rats (Fig. 3, B–D). A recent study also showed that TRPV1 participates in nociception especially under extremely acidic conditions [16].

Recent researches have shown that both TRPA1 and TRPV1 are possible targets of endogenous formaldehyde *in vitro* and *in vivo* [9]. In the report of Macpherson et al, formaldehyde-evoked calcium responses in DRG neurons and nocifensive behaviors were almost abolished in TRPA1^−/−^ mice. At the same time, formaldehyde could still evoke pain responses in the TRPA1^−/−^ mice. This suggests that formaldehyde does not merely activate TRPA1. In our present study, formaldehyde (>0.1 mM) was found to activate TRPV1 (Fig. 1A), especially in the acidic environment. We think that TRPV1 or TRPA1 are all under the mechanisms of pain. AP-18 (a TRPA1 antagonist) partially decreased formaldehyde-induced pain (pH 5.0∼6.0) and did not attenuated capsaicin-induced pain behaviors (Fig. 3B). This implies that under an acidic microenvironment of cancer tissues, TRPV1 may play a more critical role than TRPA1. Whether TRPA1 also participates in bone cancer pain is unknown, but will be investigated in our further research.

### Formaldehyde under acidic environment induces pain responses via TRPV1

With patch clamp recording, it was found that formaldehyde (>3 mM) activated TRPV1 directly in TRPV1-transfected CHO cells. While neither an acidic environment alone (pH 6.0), nor formaldehyde at low concentration alone (<3 mM) elicited currents, formaldehyde at the same low concentration under an acidic environment (pH 6.0) dose-dependently induced currents via TRPV1 (Fig. 5, A and B). In fact, formaldehyde (1∼10 mM) did not elicit currents in ASIC1a-transfected CHO cells (data not shown). These data indicate that TRPV1 (and not ASIC1a) is the direct target of formaldehyde, especially formaldehyde in an acidic environment. Formaldehyde also enhanced capsaicin-induced currents *in vitro* (Fig. 5, C and D). Formaldehyde level was elevated to about 0.6 mM in the bone morrow of this model *in vivo* (Fig. 1E), and formaldehyde (1 mM) under an acidic environment (pH 5.0) elicited C-fiber discharges *in vivo* (Fig. 5, E and F). Formaldehyde-induced pain responses in rat were obviously enhanced under an acidic environment (pH 5.0) *in vivo* (Fig. 3, A and B). It has been reported that microenvironment of tumor tissues has pH values of 4∼5 [12], and that pain behaviors could be induced at a pH as low as 5.0 through activation of ASICs and/or TRPV1 [15]. These data suggests that accumulated formaldehyde and acidic environment in tumor tissues synergistically induce pain responses by activating TRPV1 in afferent C-fiber of bone marrow or skin.

Moreover, formaldehyde up-regulated NGF expression in mast cells *in vitro*, and NGF secreted by mast cells and macrophages could up-regulate TRPV1 [2]. This implies that formaldehyde secreted by tumor tissues possibly up-regulates TRPV1 expression. Interestingly, over-expression of TRPV1 has been found in bone morrow, DRG neurons and afferent C-fibers in bone cancer pain models [1], [47]. Therefore, we hypothesize that the proliferating cancer cells secrete excessive endogenous formaldehyde in the initial stages, and then formaldehyde up-regulates TRPV1 expression in the afferent nerves. Consequently, over-expression of TRPV1 increases mechanical sensitization by decreasing pain thresholds of *patients* with cancer. Then, as the tumor progresses, acceleration of acidification and chronic accumulation of formaldehyde lead to mechanical allodynia or severe pain via ASICs and/or TRPV1 in skin or bone marrow of the cancer patients (Fig. S2).

Although, blockade of TRPV1 has been suggested as a possible therapeutic target to relieve pain [1], recent research has shown that the chronic blockade of this receptor may increase risk of cancer development [48]. In our study, we found that although capsazepine and melatonin all attenuated bone cancer pain responses, they did not decrease local formaldehyde levels in spinal cord and blood (Fig. S1, A and B). More importantly, formaldehyde can promote proliferation of cells [49], and it is a risk factor for cancer development [7]. This hints that formaldehyde may be a critical factor of the glial over-proliferation in the spinal cord of this bone cancer pain model [44]. Interestingly, melatonin has been used clinically for breast cancer [50]. We found that it inhibited acute formaldehyde- and capsaicin-induced pain behaviors (Fig. 3, A and C), as shown previously [22], [51]–[52]; and it blocked formaldehyde or capsaicin-elicited Ca^2+^ influx in DRG neurons and TRPV1-transfected CHO cells (Fig. 4). But, the melatonin receptor is not expressed in DRG neurons and CHO cells [53], [54]. Melatonin may act by antagonizing TRPV1. Potential side effects of chronic blockade of TRPV1 require further investigation.

### Formaldehyde scavengers decrease pain responses by decreasing formaldehyde level

In the classical formalin test, resveratrol (exogenous formaldehyde scavenger) [55], [56] and glutathione (endogenous formaldehyde scavenger) [57]–[59] inhibited formalin-induced pain responses (Fig. 3, C and D). To test whether resveratrol and glutathione are formaldehyde scavengers, at the molecular level, we found that resveratrol and glutathione brought about chemical deactivation of formaldehyde *in vitro* (Fig. 6B). At the cellular level, they also inhibited formaldehyde-induced neurotoxicity (Fig. 6, C and D); at the tissues level, resveratrol and glutathione attenuated MRMT-1 bone cancer pain responses in rats by decreasing endogenous formaldehyde levels in the spinal cord *in vivo* (Fig. S1A). These data further confirm that they are formaldehyde scavengers. Resveratrol inhibits proliferation of cancer cell by scavenging intracellular endogenous formaldehyde [60]. This may be the mechanism by which resveratrol defends against all kinds of cancer [61]. A previous study showed that the level of glutathione was significantly decreased in the blood of patients with breast cancer [62]. Moreover, by conferring resistance to a number of chemotherapeutic drugs, elevated levels of glutathione in tumor cells are able to protect these cells in bone marrow, breast, colon, larynx and lung cancers [63]. Both resveratrol and glutathione compounds are antioxidants. Interestingly both TRPA1 and TRPV1 are activated by oxidative stress [64], [65]. The antioxidant effect of resveratrol and glutathione may partially prevent oxidative stress-induced pain.

In summary, the present study indicates that accelerated acidification and chronically accumulated formaldehyde which are derived from local cancer tissues synergistically stimulate nerve fiber endings and lead to bone cancer pain. Use of formaldehyde scavengers may be a novel therapeutic approach for treatment of bone cancer pain.

## Materials and Methods

### Ethics statement

All experiments involving animals were conducted with the approval of the Peking University Animal Care and Use Committee. Informed consent was obtained for all participants and written by themselves. All the clinical investigation was performed after approval by the Ethics Committee of Peking University Health Science Center.

### Cancer tissues from patients suffered from bone cancer pain after clinic pain assessment

In all cases, bone cancer pain was indicated based upon clinical diagnosis. A questionnaire which included a diagram to indicate painful and tender areas and the pain descriptors from the McGill Pain Questionnaire were produced, along with a 10-cm unmarked visual analogue scale (VAS), and VAS scores marked by the patient in centimeters being more than zero to identify those patients with breast pain [66]. Nineteen breast cancer pain patients and 6 normal women carried out the clinical pain assessment by doctors. Tumor samples from 19 breast cancer patients and clinic data were provided by the Department of General Surgery, Peking University Third Hospital. Tissues adjacent to breast cancer were adipose tissue and were not used in this study. Tumor samples from 10 lung cancer patients which included adenocarcinoma, squamous cell carcinoma and pulmonary lymphoma were provided by the Department of Thoracic Surgery, Peking University People's Hospital, tissues adjacent to cancer were obtained from 4 lung cancer patients. Both cancer tissues and tissues adjacent to cancer were frozen immediately with and stored in liquid nitrogen until they were used for evaluation of formaldehyde concentration.

### Bone cancer pain rat model

A rat bone cancer pain model was established using Sprague-Dawley rats with MRMT-1 rat mammary gland carcinoma in a manner similar to that in a previous report [67]. After anesthesia, the tibia was carefully exposed and a 23-gauge needle was inserted into the intramedullary canal of the bone. It was then removed and replaced with a long thin blunt needle attached to a 10-µl Hamilton syringe containing carcinoma cells. A volume of 4 µl containing MRMT-1 cancer cells (4×10^4^), heat-killed cancer cells or phosphorylated buffer solution (PBS) was injected into the bone cavity. Following injection, the entry site on the bone was sealed with bone wax. Doses of test reagents were given at 9, 11, 13 and 15 day respectively, including capsazepine (intravenous injection through the tail or intraperitoneal injection, *i.p.*) and resveratrol (*i.p.*). These regents were dissolved in DMSO (final concentration <10%). Glutathione (*i.p.*) was dissolved in normal saline. All reagents were obtained from Sigma, unless otherwise indicated. The bone marrow and spinal cords of these rats were taken out for formaldehyde measurement with HPLC.

To assess the bone destruction after inoculation, tibial bone radiographs from both hind limbs on 7 and 15 days were taken with a Digital Radiographer System (E-COM Technology Co. Ltd., Guangdong, China). Radiological scores were given based on careful, blind analysis of radiographs, taken from the ipsilateral and contralateral legs of MRMT-1-treated, heat-killed MRMT-1-treated, vehicle-treated and naive rats (n = 4 for each group). Scores were given as in previous report [44]. All scores related to the tibia (bone): 0, normal bone structure without any sign of deterioration; 1, small radiolucent lesions in the proximal epiphysis, close to the site of the injection; 2, increased number of radiolucent lesions, loss of medullary bone; 3, loss of medullary bone, plus erosion of the cortical bone; 4, full thickness unicortical bone loss; 5, full thickness bicortical bone loss and displaced fractures.

### Hot plate test for thermal hyperalgesia

Male Sprague-Dawley rats (150∼200 g) were provided by the Department of Animal Science of Peking University. Animals were raised under natural diurnal cycles and had free access to water and food. They were habituated to the testing paradigms for 3∼5 days before experiment. Animal treatment was in compliance with the Guidelines of the International Association for the Study of Pain [68]. On days 1, 3, 7, 11 and 15 after injection of MRMT-1 cells, heat-killed cells or PBS injection, thermal hyperalgesia was tested with hot plate. Rats were habituated to the experimental environment for 30 min in their home cage. Rats were placed on the hot plate (52±0.5°C) and the interval time until the rat jumped or licked either of its hind paws was recorded as hot plate latency. Following a response, the rat was immediately removed from the plate. Each test was repeated three times with a 15 min interval between tests [69].

### Von Frey hair test for mechanical allodynia

Each animal was placed in a clear Plexiglas compartment with a mesh floor and was allowed to habituate for 20 min. On days 1, 3, 7, 11 and 15 after injection of MRMT-1 cells, heat-killed cells or PBS, mechanical allodynia was evaluated with application of *von* Frey hair (Semmes-Weinstein Monofilaments, North Coast Medial Inc., San Jose, CA) in ascending order of force (0.41∼15.1 g) to the plantar surface of the hind paw. The rat was placed in the test box and allowed to settle in for 5∼10 min. An ascending series of *von* Frey hairs with logarithmically incremental stiffness (0.40, 0.60, 1.4, 2.0, 4.0, 6.0, 8.0, and 15.0 g) were applied perpendicular to the mid-plantar surface (avoiding the less sensitive tori) of each hind paw. Each *von* Frey hair was held about 1∼2 s, with a 10-min interval between each application. A trial began with the application of the 2.0 g *von* Frey hair. The positive response was defined as a withdrawal of hind paw upon the stimulus. Whenever a positive response to a stimulus occurred, the next lower *von* Frey hair was applied, and whenever a negative response occurred, the next higher hair was applied. The testing consisted of five more stimuli after the first change in response occurred, and the pattern of response was converted to a 50% *von* Frey threshold using the method as previously reported [70].

### Classic formalin-induced spontaneous pain (formalin test)

Following a 30-min habituation to the observation cage, male Sprague-Dawley rats (300∼350 g) received an *s.c.* injection of 50 µl of a 5% formalin (1662 mM formaldehyde) solution into the dorsal aspect of the right hind paw. Nociceptive behaviors which were recorded included flinching, licking or biting the injected paw as previously described [22], [51]. Test reagents with or without formalin was injected into paw plantar aspect of the paw. Capsazepine, melatonin, AP-18 and resveratrol were dissolved in DMSO (final concentration <10%); formalin and glutathione were dissolved in normal saline.

### Formaldehyde test for acute nociception

Following a 20 min adaptation, rats received a subcutaneous injection of formaldehyde (0.1∼100 mM) at pH 5.0 or 6.0 (mimicking a moderate to severely acidic tumor microenvironment) into the plantar of right hind paw using a microsyringe with a 26-gauge needle. The length of time that the animals spent flinching, lifting, licking or biting the injected paw was recorded with a chronometer, and was considered as an indicator of pain response in the early phase (0∼5 min) as previously described [46]. Agonists or antagonists (100 µl/paw) which were injected included capsazepine, AP-18 and melatonin which were dissolved in DMSO (final concentration <10%). Formaldehyde was dissolved in normal saline.

### Cell culture

Chinese hamster ovary (CHO) cells were cultured in Ham's F-12 medium. MRMT-1 rat mammary gland carcinoma cells (Novartis Oncology Research, Basel) and H1299 human lung cancer cells were cultured in RPMI 1640 (Gibco) medium, human SY5Y cell lines were cultured in Dulbecco's modified Eagle's medium (DMEM). All culture dishes were kept in an incubator under a humidified atmosphere (37°C, 95% air and 5% CO_2_).

Dorsal root ganglion (DRG) neuron culture was carried out following a modification of a previously described procedure [71]. Briefly, DRG from 3-week-old SD rats were digested with trypsin/EDTA solution for 40 min and dissociated in growth medium containing DMEM, 5% inactivated fetal bovine serum, 5 mM glutamine, B27 supplement (GIBCO), 100 ng/ml of nerve growth factor (Becton Dickinson) and 0.6% dextrose. Cells were seeded at a density of 5×10^5^ per well. After 2 days, cultures were treated with 10 µM cytosine arabinoside to control growth of dividing cells. Cells were used one week after culture.

### Cell viability assayed with MTT

MTT solution was prepared in complete medium to a concentration of 1 mg/ml just before use. Cells were diluted in fresh complete medium and seeded in 96-well plates. After allowing overnight attachment, cells were treated with various concentrations of formaldehyde or reagents for 4 h. Optical density were determined by a spectrophotometer.

### Formaldehyde measurement with high performance liquid chromatography with fluorescence detection (Fluo-HPLC)

Media from the cultured cancer cell lines, bone marrows, the homogenate of spinal cords of model rats and cancer patient tissue specimens were harvested for formaldehyde assay. An HP 1100 HPLC Instrument (Hewlett-Packard, USA) with a fluorodetector was used. The method was as described in our previous report [72].

### Cytosolic Ca^2+^ concentration in TRPV1-transfected CHO cells and DRG neurons

For transfection of CHO cells with TRPV1 plasmids, CHO cells were harvested after brief trypsin digestion and seeded onto confocal plate chambered cover-glasses (LabTek, Nunc) precoated with 20 µg/ml poly-L-lysine. Actively growing cells were transfected with a pcDNA3.1-TRPV1 plasmid by lipofectamine plus (Life Technologies, Rockville, MD, USA). Control cells were transfected with pcDNA3.1 plasmid only. After 16∼24 h culture, cells were challenged with formaldehyde or capsaicin. DRG neurons were freshly isolated with trypsin and collegenase digestion as in our previous report [73].

TRPV1-CHO cells and DRG neurons were loaded with 50 µM Fluo-3/AM (Molecular Probes) and incubated for 30 min at 37°C. Fluo-3/AM was excited at 488 nm, and its emitted fluorescence was collected at 515 nm. The control bath solution (pH 7.4) was used as described previously [69]. Changes in cytosolic [Ca^2+^]i concentration in the TRPV1-CHO cells and in the DRG neurons were measured with a confocal laser scanning microscope (Leica Company Ltd., Germany).

### Whole cell patch-clamp recording of TRPV1 current *in vitro*

Recordings in TRPV1-CHO cells were made with standard whole-cell patch-clamp method as described in our previous report [73]. Experiments were carried out at room temperature (22∼24°C). The extracellular solution consisted of 130 mM NaCl, 5 mM KCl, 2 mM CaCl_2_, 1 mM MgCl_2_, 30 mM glucose and 25 mM HEPES-NaOH and the pH was adjusted to 7.3. Patch pipettes (resistance 2∼5 MΩ) were filled with 140 mM CsCl, 4 mM MgCl_2_, 10 mM EGTA and 10 mM HEPES-CsOH (pH 7.3). Reagents were applied with an automated perfusion device.

### Extracellular electrophysiological recording of C-fiber firing *in vivo*

Male Sprague-Dawley rats weighing 300∼350 g were anesthetized with urethane (1.5 g/kg, *i.p.*). Animal preparation and the teased fiber recording method were carried out as previously reported [73]. Briefly, the L5 dorsal root was exposed by lumbar laminectomy and covered with warmed (36°C) paraffin oil. Fine axon bundles (microfilaments), cut centrally but in continuity with the DRG distally, were separated from the dorsal root near its point of entry into the spinal cord. C-fiber was recorded for 2 min to detect its receptive field and to confirm its conduction velocity (less than 2.0 m/s). C-fiber firing within the following 5 min was taken as the basal firing. After injection of 1 mM formaldehyde with pH 5.0 (mimicking an extremely acidic tumor microenvironment) or vehicle only (normal saline, pH 5.0) into the receptive field of a C-fiber, firing was recorded for another 5 min. The frequency of the C-fiber firing before and after formaldehyde injection was compared.

### Data analysis

Data are expressed as mean ± S.E.M. Data from *in vitro* experiments were analyzed using the Student's t test for comparison of independent means. *In vivo* data were analyzed by analysis of variance (ANOVA) followed by Dunnett's *post hoc* test repeated measures of ANOVA, and post hoc tests including Student's t test (ex vivo test). *p* values less than 0.05 were considered statistically significant.

## Supporting Information

Figure S1 Formaldehyde concentration in bone cancer pain model rats in (A) spinal cord and (B) blood. In MRMT-1 pain model of rats, formaldehyde (FA) concentration increased. TRPV1 antagonists capsazepine (CPZ 0.1 mg/ml) and melatonin (MT, 5 mg/ml) had no obvious influence on formaldehyde concentration, but formaldehyde scavengers resveratrol (Res, 0.4 mg/ml) and glutathione (GSH, 25 mg/ml) decreased FA concentration. * p<0.05, ** p<0.01; # p<0.05, ## p<0.01, all compared with PBS groups. n = 10.
(0.33 MB TIF)

Figure S2 A putative scheme that excessive formaldehyde secreted by tumor tissues and its induction on bone cancer pain under an acidic microenvironment.
(4.24 MB TIF)
