# Supplementary figures and images for: Tumor Tissue-Derived Formaldehyde and Acidic Microenvironment Synergistically Induce Bone Cancer Pain
Source: PLoS One. 2010 Apr 21;5(4):e10234. doi: 10.1371/journal.pone.0010234 (PMC2858155; doi:10.1371/journal.pone.0010234)

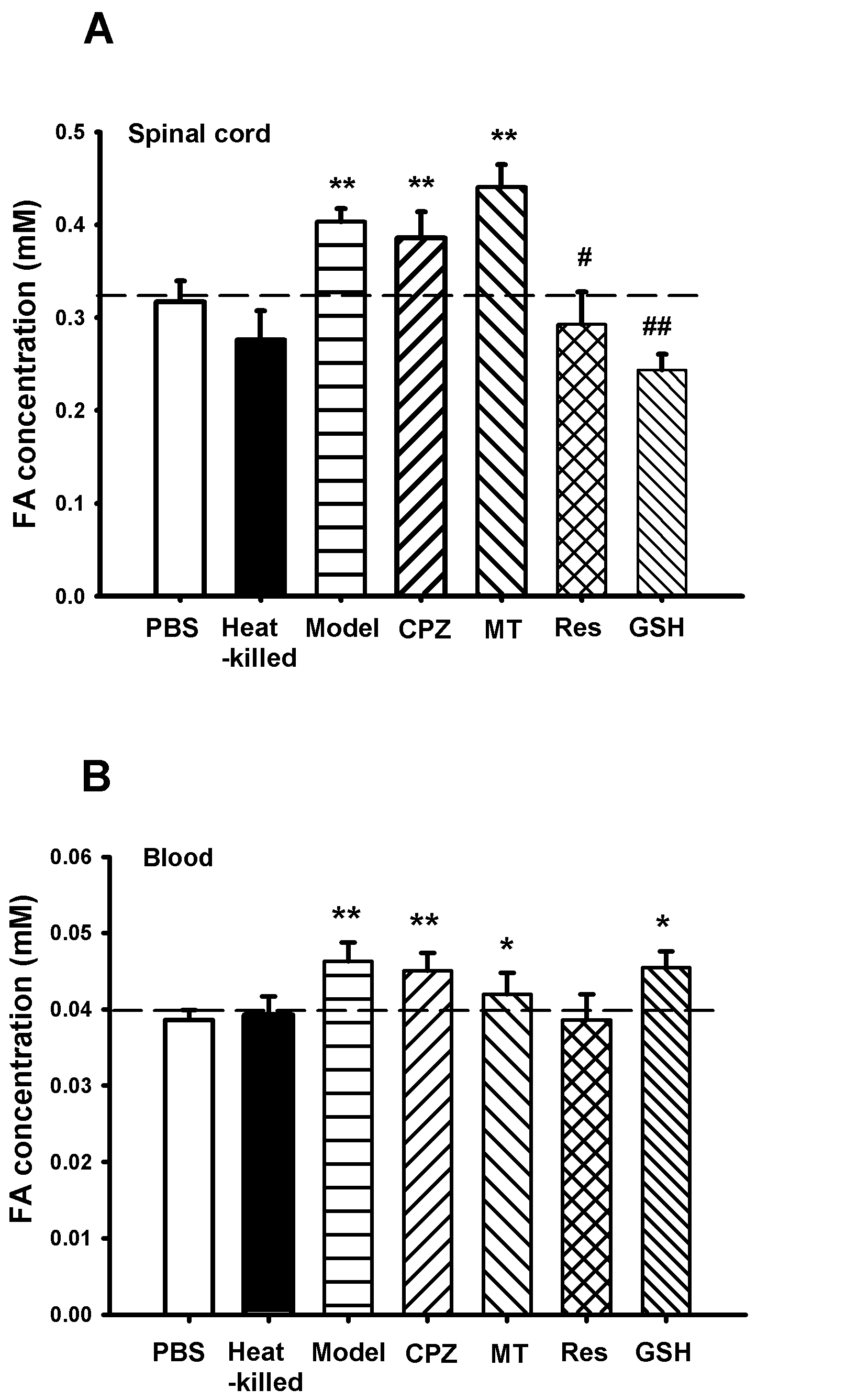

Supplement: Figure S1 — Formaldehyde concentration in bone cancer pain model rats in (A) spinal cord and (B) blood. In MRMT-1 pain model of rats, formaldehyde (FA) concentration increased. TRPV1 antagonists capsazepine (CPZ 0.1 mg/ml) and melatonin (MT, 5 mg/ml) had no obvious influence on formaldehyde concentration, but formaldehyde scavengers resveratrol (Res, 0.4 mg/ml) and glutathione (GSH, 25 mg/ml) decreased FA concentration. * p<0.05, ** p<0.01; # p<0.05, ## p<0.01, all compared with PBS groups. n = 10. (0.33 MB TIF) [file pone.0010234.s001.tif]

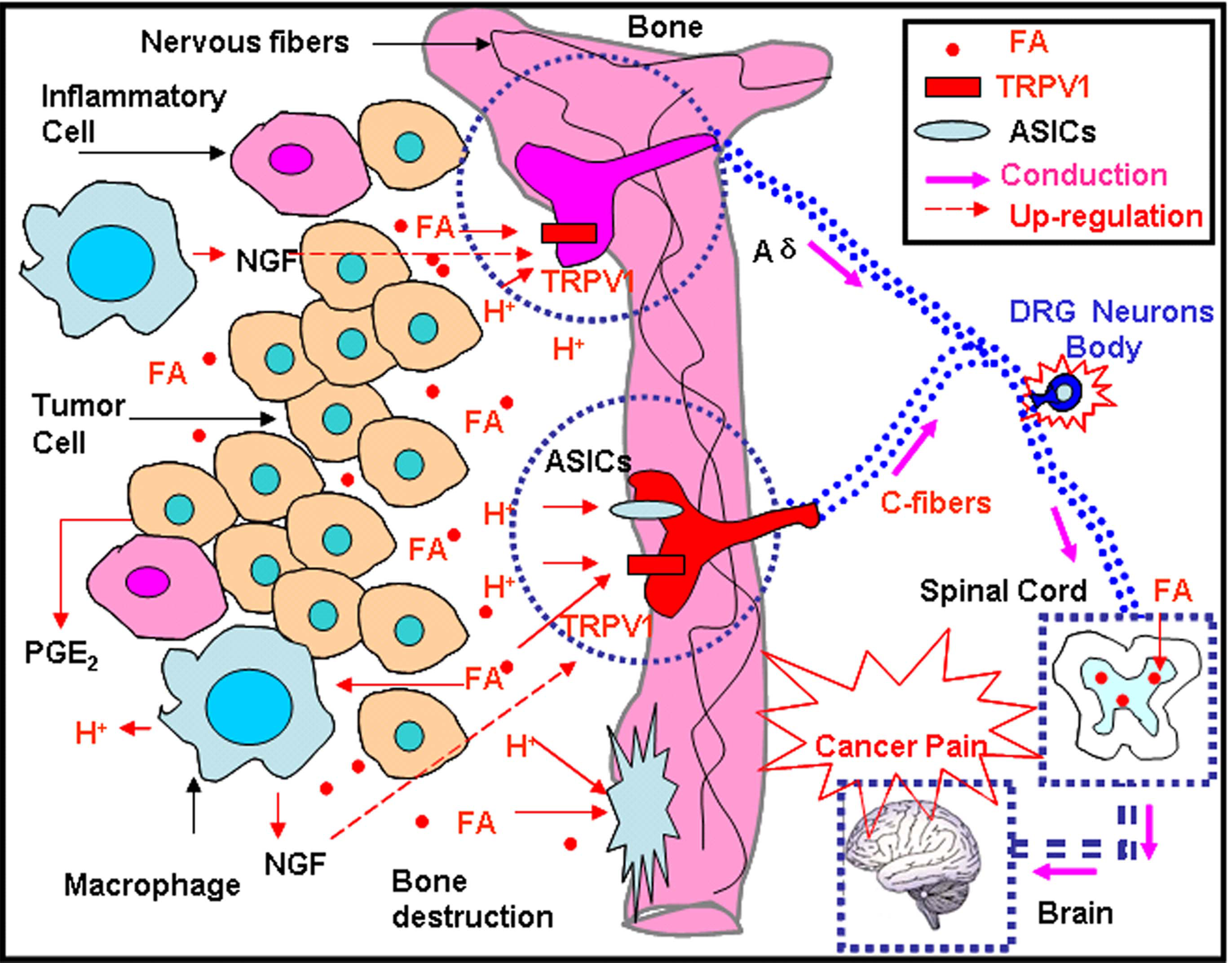

Supplement: Figure S2 — A putative scheme that excessive formaldehyde secreted by tumor tissues and its induction on bone cancer pain under an acidic microenvironment. (4.24 MB TIF) [file pone.0010234.s002.tif]
